# The Effect of Fluid Availability on Consumption and Perceptual Measures during Aerobic Exercise

**DOI:** 10.3390/ijerph20021310

**Published:** 2023-01-11

**Authors:** Courteney L. Benjamin, Luke W. Dobbins, Shealyn G. Sullivan, Rebecca R. Rogers, Tyler D. Williams, Mallory R. Marshall, Christopher G. Ballmann

**Affiliations:** 1 Department of Kinesiology, Samford University, Birmingham, AL 35226, USA; 2 SHP Research Collaborative, University of Alabama at Birmingham, Birmingham, AL 35226, USA

**Keywords:** hydration, nutrition, performance

## Abstract

Fluid availability may alter drinking behavior; however, it is currently unknown if the availability of fluid impacts behavior and gastrointestinal issues (GI) that are often associated with increased fluid intake. The purpose of this study was to determine if ad libitum (AL) versus periodic (PER) fluid intake influences fluid consumption and GI distress during exercise in trained athletes. Male and female Division I NCAA Cross Country athletes (n = 11; age = 20 ± 1 years) participated in this counterbalanced crossover study. Each participant completed a moderate intensity 10 km run on two separate occasions. In one trial, participants had unlimited availability to fluid to consume AL. In the other trial, participants consumed PER fluid at stations placed every 3.2 km. Assurance of euhydration prior to each trial was confirmed via urine specific gravity (USG) and urine color. Subjective perceptions of thirst and gastric fullness were assessed pre- and post-exercise via Likert questioning and a visual analog scale, respectively. Participants started each trial euhydrated (AL = 1.009 USG ± 0.009; PER = 1.009 USG ± 0.009; urine color AL, 3 ± 1; urine color PER, 2 ± 1). Fluid volume consumption was significantly higher during the AL condition compared to PER (*p* = 0.050). Thirst significantly increased from pre- to post-run regardless of treatment (*p* < 0.001); however, there was no significant difference between the groups (*p* = 0.492). Feelings of fullness did not change pre-post trial (*p* = 0.304) or between trials (*p* = 0.958). Increased fluid availability allows for increased fluid consumption without the negative experience of GI discomfort.

## 1. Introduction

Hydration and fluid balance have been repeatedly shown to alter cognitive and physical performance in general and athletic populations alike [1,2]. Euhydration has been shown to produce positive effects in competition [3,4,5]. There are three well-known strategies to consider when determining how an athlete should hydrate during competition: prescribed fluid intake, ad libitum (AL) fluid intake, and drinking to thirst. Prescribed fluid intake refers to consuming fluid needed to replace sweat loss <2% of body mass loss [6]. AL fluid intake refers to consuming fluid at whatever volume is desired. Drinking to thirst refers to consuming fluid based on how thirsty one feels [5,6,7]. Several argue that drinking to thirst is the optimal strategy for performance due to its ability to maintain a reasonable hydration status with the lack of gastrointestinal (GI) discomfort that can be associated with prescribed fluid consumption, as well as to prevent hyponatremia [5,8,9]. Others argue that drinking to thirst still results in significant fluid losses known to negatively impact performance and that prescribed fluid intake is the only way to optimize performance [6,7,10]. AL fluid intake may be a reasonable solution to maintain hydration status during exercise while limiting GI distress. Although AL fluid intake and drinking to thirst share many similarities, AL fluid intake relies heavily on the availability of the fluid rather than the perception of being thirsty [11]. Additionally, AL fluid consumption has been shown to be impacted by flavor, temperature, and composition, as well as encouragement to drink, pauses in training, and training duration [12,13]. Altogether, it is still unclear how the availability of fluid during exercise impacts thirst perception, GI distress, and fluid intake with AL fluid consumption.

Maintenance of a euhydrated state during aerobic exercise, even in the absence of thirst, has been previously linked to optimal performance [3]. For example, Adams et al. demonstrated an increase in power output and speed during a 2 h cycling test amid a euhydrated trial compared to a hypohydrated trial, even when participants were blinded to their hydration state [3]. Thus, hydration plays a pivotal role in aerobic exercise performance, which may be autonomous or independent of thirst. Fluid consumption during a field-based study demonstrated the impact of prescribed fluid intake on the volume of fluid consumed. Dion et al. showed in their study of half marathon runners that athletes who followed a prescribed fluid intake protocol consumed significantly more fluid, had a lower heart rate, and reported lower thirst levels compared to athletes without a prescribed fluid intake protocol [5]. In another study, Armstrong et al. examined two distinct groups during a 164 km cycle race comparing AL fluid intake and drinking to thirst, and showed no significant difference in the volume of fluid consumed between groups. This is believed to be a result of similar psychological and perceptual responses to both patterns of drinking [11]. Thus, whether participants drank AL or drank to thirst, the overall fluid intake was similar and did not have a significant impact on the results. 

Regardless of the fluid intake method, fluid availability may strongly influence the fluid intake of exercising individuals [14]. Aragón-Vargas et al. discussed the impacts of availability on fluid intake in professional soccer players [15]. In this study, hydration level was assessed pre- and post-match. Hypohydration was observed and credited to: (1) minimal opportunities for fluid intake and (2) limited fluid volume consumed due to gastric emptying and intestinal absorption rate. While the type of competition may influence fluid availability, this concept is particularly interesting for the runner who has the option to have fluid readily available during training but chooses not to during races. These factors, along with the known benefits of maintaining a euhydrated state during exercise, lead to questions regarding what exactly influences fluid intake behavior. 

While research has established that fluid consumption behavior is multi-faceted, it has not addressed how the availability of fluid may impact fluid intake during exercise [5]. Although there has been immense research on hydration and fluid consumption, there is little literature that expands upon the importance of fluid availability and its impacts on thirst perception and GI distress. Understanding how availability plays a role in fluid intake behavior could help foster innovative ideas on how aerobic performance can be improved with proper fluid availability. Therefore, the purpose of this study was to determine if AL versus periodic (PER) fluid intake influences fluid consumption and GI distress during exercise. We hypothesize that AL will result in the consumption of higher fluid volume, less perceived thirst, and less GI discomfort than PER. 

## 2. Materials and Methods

### 2.1. Participants

A group of 11 (5 males and 6 females) non-heat-acclimatized NCAA Division 1 Cross Country athletes participated in this study (mean ± SD; age, 20 ± 1 y; height, 174 ± 10 cm; body mass, 142.3 ± 16.0 kg). Participants were assumed to be non-heat-acclimatized due to the time of year that the study took place (February–March) in the United States. To ensure the health and safety of the participants, a health history and Physical Activity Readiness Questionnaire (PARQ) was completed prior to involvement. All participants met the ACSM guidelines for being physically active (at least 3 days per week of at least 30 min of moderate intensity activity for at least 3 months), had not experienced a lower-body injury within the past 6 months from the date of the first visit, nor did they indicate cardiovascular, metabolic, or renal disease. For each visit, participants were asked to refrain from consuming alcohol, caffeine, nicotine, and pre-/post-workout supplements for 12 h prior to each visit, and to avoid strenuous exercising for at least 24 h prior to the time of each scheduled visit. The experimental protocol was approved by the Samford University Review Board (#EXPD-HP-21-S-1) and participants provided their written informed consent prior to participation, in accordance with the Declaration of Helsinki. 

### 2.2. Study Design

**Figure 1:**
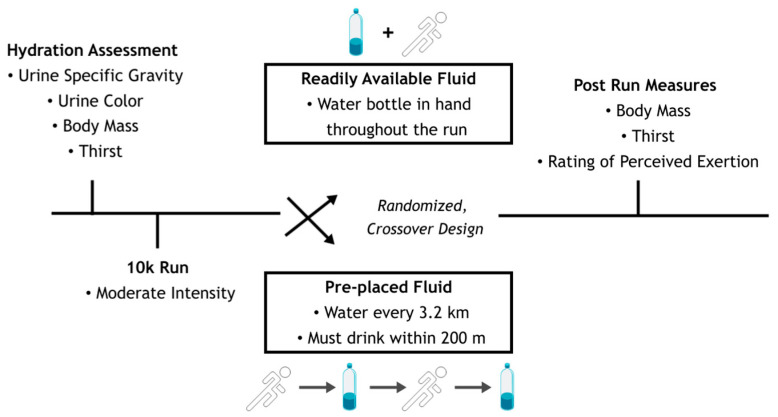
Fluid availability study design.

In a randomized, counterbalanced, crossover study design, participants completed two trials: (1) 10 k run with readily available fluid (AL) and (2) 10 k run with pre-placed fluid (PER) (Figure 1). Prior to both trials, participants were instructed to consume 500 mL of fluid the night before the trial and 500 mL of fluid within 2 h before the trial began to ensure euhydration. At the beginning of the first trial, height, body mass, and age were recorded. Each trial took place over a designated 10 k course. Participants were instructed to maintain a similar, moderate pace for both trials. Each trial was separated by at least 1 week and were postponed during times of extreme weather conditions (extreme heat, lightning, etc.). The ambient temperature (25.2 ± 1.9 °C), relative humidity (42.2 ± 7.7%), and wind speed (1.3 ± 0.5 mph) were similar between the trials. 

In one trial, participants were given a fluid bottle filled with water to carry with them throughout the duration of the run (AL). To ensure participants always had availability of fluid, additional fluid bottles were placed throughout the course for the participants to use if their initial bottle had become low on fluid. In the other trial, participants were able to consume fluid at fluid stations placed every 3.2 km throughout the course (PER). Participants were given the option to grab a fluid bottle filled with water at the stations and consume fluid for a maximum of 200 m from the fluid station while running. 

### 2.3. Study Variables

For all trials, participants were asked to confirm their adherence to the instructed pre-trial fluid intake protocol and major deviations from the protocol resulted in the trial being rescheduled. Upon arrival at all trials, hydration was assessed through urine analysis. Participants provided a urine sample upon arrival and urine specific gravity (USG) was assessed using a hand-held refractometer (Ketotek, Hangzhou, China) and urine color was assessed through comparison with a urine color chart (Hydration Check, Human Hydration, VA, USA). Euhydration was defined as a USG of less than 1.020 [16]. If USG was between 1.020 and 1.025, participants consumed approximately 500 mL of fluid prior to starting exercise. If the USG was greater than 1.025, the trial was rescheduled for another day. Participants were instructed to wear similar athletic clothing for each trial. 

Before beginning and immediately following exercise, nude body mass (WB800S Tanita, IL, USA), thirst, and rating of perceived exertion were assessed. To assess nude body mass, participants entered a private and locked room where they removed all clothing and dried off with a towel (after exercise) to obtain a body mass. Thirst was assessed by asking the participants to rate their sensation of thirst on a 9-point Likert scale with 1 indicating “Not thirsty at all” and 9 indicating “Very, Very Thirsty” [17]. Additionally, a visual analog thirst assessment was completed [18]. This survey consisted of the following questions: “How thirsty do you feel right now?” (not at all thirsty–very thirsty) [Thirst]; “How pleasant would it be to drink some fluid right now?” (very unpleasant–very pleasant) [Satiety]; “How dry does your mouth feel right now?” (not at all dry–very dry) [Dryness]; “How would you describe the taste in your mouth?” (normal–very unpleasant) [Taste]. This VAS also included two specific questions related to GI discomfort: “How full does your stomach feel right now?” (not at all full–very full) [Fullness]; “How sick to your stomach do you feel right now?” (not at all sick–very sick) [Nausea]. 

Rate of perceived exertion was recorded according to a Borg scale (6–20). Average heart rate and total duration were monitored in both trials using wrist-worn GPS and heart rate devices (Garmin Forerunner 245, Olathe, KR, USA). The trials took place over three different days at the same time of day (1500–1700) to accommodate the participants’ schedules. Environmental conditions were assessed at the beginning of each trial using the Kestrel 3000HS Heat Stress Meter (Kestrel, Boothwyn, PA, USA).

### 2.4. Statistical Analysis

All statistical analyses were completed using Jamovi (The jamovi project (2020) Jamovi (Version1.2)). Paired samples *t*-tests were used to compare hydration indices (USG, urine color, body mass, and sweat loss) and HR between trials. Two-way repeated measures ANOVAs (trial: 2 levels, time: 2 levels) were used to assess perceptual measures. Tukey correction was used for post hoc analyses. Alpha was set at *p* ≤ 0.05, a priori. Data were reported as mean ± standard deviation, Cohen’s d effect sizes (ES) for *t*-tests, and partial eta squared effect sizes (ηp^2^) for ANOVAs. Cohen’s d was interpreted according to the following thresholds: <0.2 = trivial, 0.2–0.6 = small, 0.7–1.1 = moderate, 1.2–2.0 = large, and >2.0 = very large [19]. ηp^2^ was interpreted according to the following thresholds: 0.01 = small effect, 0.06 = medium effect, and 0.14 = large effect [20].

## 3. Results

### 3.1. Hydration, Heart Rate, and Duration

**Figure 2:**
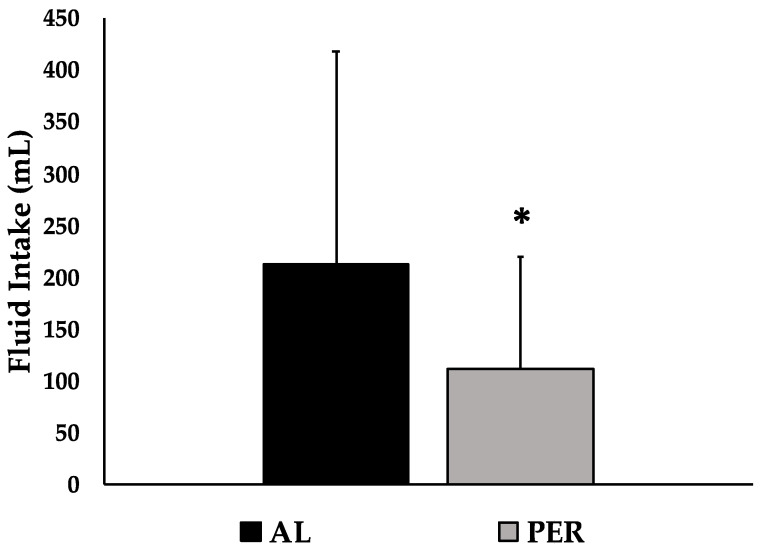
Fluid consumption between trials. AL = ad libitum fluid intake while carrying fluid bottle throughout exercise. PER = fluid intake only at stations placed every 3.2 km. Data are presented as mean ± standard deviation. * *p* ≤ 0.05 indicates statistically significant differences between trials.

USG, urine color, % body mass loss, HR, and duration were not significantly different between trials (Table 1). Figure 2 demonstrates that the amount of fluid consumed in AL (242.7 ± 209.0 mL) was significantly greater (*p* = 0.05; ES = 0.67) than the amount of fluid consumed in PER (111.4 ± 110.0 mL).

### 3.2. Thirst and Gastrointestinal Issues VAS Scale

The descriptive results from the visual analog thirst scale are recorded in Table 2. Thirst, satiety, dryness, and taste showed a significant difference from the beginning to end of each trial (Thirst: *p* < 0.001, ηp^2^ = 0.76; Satiety: *p* < 0.001, ηp^2^ = 0.85; Dryness: *p* < 0.001, ηp^2^ = 0.79; Taste: *p* = 0.03, ηp^2^ = 0.41). Thirst did not demonstrate differences between trials (*p* = 0.21, ηp^2^ = 0.15) and there was no interaction effect (*p* = 0.17, ηp^2^ = 0.18). Satiety did not demonstrate differences between trials (*p* = 0.79, ηp^2^ = 0.01) and there was no interaction effect (*p* = 0.19, ηp^2^ = 0.17). Dryness did not demonstrate differences between trials (*p* = 0.48, ηp^2^ = 0.05) and there was no interaction effect (*p* = 0.62, ηp^2^ = 0.03). Taste did not demonstrate differences between trials (*p* = 0.76, ηp^2^ = 0.01) and there was no interaction effect (*p* = 0.53, ηp^2^ = 0.04). There was no difference between time (*p* = 0.30, ηp^2^ = 0.02), trial (*p* = 0.96, ηp^2^ = 0.00), or an interaction effect for fullness (*p* = 0.64, ηp^2^ = 0.00). There was no difference between time (*p* = 0.75, ηp^2^ = 0.01) for nausea and there was a significant difference between trial (*p* = 0.02, ηp^2^ = 0.43). However, there was no significant interaction for nausea (*p* = 0.80, ηp^2^ = 0.01).

### 3.3. RPE and Thirst Results

RPE was significantly higher from pre-post trial in both trials (AL-pre = 6 ± 1, AL-post = 12 ± 2, *p* < 0.001; PER-pre = 6 ± 2, PER-post = 13 ± 2, *p* < 0.001, ηp^2^ = 0.92); however, there were no differences between trials (*p* = 0.42, ηp^2^ = 0.07) and there was no interaction effect (*p* = 0.65, ηp^2^ = 0.02). Thirst from the 1–9 scale was significantly higher from pre-post trial in both trials (AL-pre = 3 ± 1 AL-post = 5 ± 1; PER-pre = 3 ± 1 PER-post = 5 ± 2, *p* < 0.001, ηp^2^ = 0.82); however there were no differences between trials (*p* = 0.49, ηp^2^ = 0.05) and there was no interaction effect (*p* = 0.12, ηp^2^ = 0.24). 

## 4. Discussion

The objective of this study was to determine the impact of fluid availability on fluid intake behavior and perceptual thirst and GI discomfort measures during moderate intensity exercise. The main finding of the current study demonstrates that increasing fluid availability when drinking AL resulted in significantly greater fluid intake over a 10 km run. Importantly, the increase in fluid volume did not result in an increase in GI discomfort, indicating that AL fluid availability may be a practical and ideal hydration strategy during exercise. The literature has established that hypohydration can negatively influence aerobic exercise performance [3,21,22]. Optimal fluid intake protocols depend on several factors, such as the exercise duration and intensity, goal, and environmental conditions [6]. One argument against using prescribed fluid intake is the potential implications on GI discomfort or increased risk of experiencing hyponatremia [9]. Drinking to thirst, especially when fluid is not readily available, has been shown to result in lower volumes of fluid intake [23]. 

Current results indicate that the amount of fluid consumed had no significant effect on the sensation of thirst or GI discomfort. This is contrary to previous studies that showed a decreased sensation of thirst and increased abdominal discomfort when participants drank more fluid during exercise [5]. However, this finding may be due to the difference in the total volume of fluid consumed. In the referenced study, participants consumed approximately 3.5× as much fluid in the prescribed fluid trial compared to drinking to thirst. In the present study, participants drank twice the volume of fluid when the fluid bottle was readily available compared to when they could only consume fluid at stations. Another potential reason for no difference in GI distress between trials is the moderate intensity and short duration of the exercise in the present study. GI distress is not common in shorter exercise durations at lower intensities [24]. These findings highlight the potential benefit of AL fluid intake when fluid is readily available and the importance of understanding how human behavior can influence hydration practices.

Armstrong et al. summarize thirst behavior into two main categories that affect thirst: homeostatic thirst and non-homeostatic thirst [14]. Homeostatic thirst is related to biological mechanisms that affect thirst, such as a change in plasma volume affected by sweat rate [14]. Non-homeostatic thirst is influenced by social experiences such as learned behaviors, dry mouth, palatability of fluid, etc. [14]. This review further explains how this mechanism functions in the brain. Specifically, the oral pharyngeal organs stimulate the lamina terminalis in our brain in response to these internal and external mechanisms to indicate thirst. When we consume fluid, our subfornical organ inhibits the stimuli to the brain, causing one to feel satiated [25]. Although both groups in the present study reported similar levels of satiety and dryness before and after exercise, the AL group clearly consumed more fluid, highlighting the influence of the non-homeostatic mechanism of fluid availability on fluid intake behavior. 

Another factor that may influence fluid consumption behavior is the gastric emptying rate. As fluid is ingested, the pyloric sphincter regulates the rate at which fluid leaves the stomach and enters the rest of the GI tract. The timing and volume of fluid consumption can exceed the stomach’s capacity to distribute fluid, thus, resulting in discomfort and pain. Because of this, even though the sensation of thirst may be present, gastric emptying rates only allow for the consumption of a limited amount of fluid before the stomach feels full [26]. This is one mechanism that helps explain why participants consumed less fluid when fluid was placed at stations. When participants had fluid bottles readily available to them, they could sip fluid whenever they desired, not filling their stomach at once with fluid. When the fluid was only available at the stations, participants had to decide how much fluid they should consume to hydrate without causing GI discomfort. In all, consuming fluid AL appeared to provide more opportunity for fluid consumption without the negative consequences of GI discomfort. 

The previous literature has indicated that maintaining a body mass loss <2% is ideal for optimal exercise performance [1]. In the current study, despite vastly different consumption between groups, neither group lost >2% of their body mass. Our study demonstrated that for a moderate paced 10 km run, having fluid stations placed 3.2 km apart is enough to keep collegiate runners in a euhydrated state in these environmental conditions if they start the run euhydrated. However, since the amount of fluid consumed was different between the groups, we hypothesize that the fluid consumed from stations every 3.2 km may not be enough to replace the fluid lost via sweat to remain in a euhydrated state if the distance, duration, intensity, or environmental conditions were increased. One limitation to this study is that we did not examine the impact that higher intensity or longer duration exercise would have on fluid intake behavior or GI discomfort. Future research could examine the fluid intake behaviors and GI discomfort of these two strategies in longer distances such as the half marathon and marathon or the same distance at a higher exercise intensity. Another limitation to the current study is performance was not assessed. To answer this specific research question, participants were instructed to run at a moderate intensity exercise since fluid intake behavior will most likely be impacted by exercise intensity. Future research should examine the impact that various fluid intake strategies may have on exercise performance. 

## 5. Conclusions

In conclusion, this study provides novel evidence that fluid intake during exercise is influenced by more than just the perception of thirst. Fluid availability is a potent influence of fluid intake behavior and is highly likely to contribute to how and when exercising individuals hydrate. AL fluid intake with readily available fluid resulted in significantly greater fluid consumption compared to fluid intake from stations, without increased GI discomfort. In order to optimize hydration during exercise, individuals should consider carrying a water source with them. This strategy may be particularly impactful when exercising in a hot climate when sweat rates are elevated.

## Figures and Tables

**Table 1:** Hydration and 10 k data.

	Mean ± Standard Deviation			
	AL	PER	*p*-Value	Effect Size
Urine Specific Gravity	1.009 ± 0.008	1.009 ± 0.009	0.79	0.08
Urine Color	3 ± 1	2 ± 1	0.22	0.39
Body Mass Loss (%)	1.4 ± 0.6	1.3 ± 0.6	0.59	0.17
Heart Rate (bpm)	157 ± 11	160 ± 11	0.22	0.40
Exercise Duration (min)	43.18 ± 0.09	42.72 ± 0.12	0.41	0.26

**Table 2:** Visual analog thirst questionnaire (mm).

	AL		PER	
	Pre	Post	Pre	Post
How thirsty do you feel right now?	1.8 ± 1.5	4.2 ± 2.5	1.7 ± 1.6	5.6 ± 2.7
How pleasant would it be to drink some fluid right now?	4.6 ± 2.0	6.8 ± 2.3	3.9 ± 2.4	7.2 ± 2.8
How dry does your mouth feel right now?	2.1 ± 1.9	4.8 ± 2.1	2.3 ± 2.3	5.6 ± 2.7
How would you describe the taste in your mouth?	0.6 ± 0.8	2.5 ± 2.3	0.9 ± 1.3	2.4 ± 2.4
How full does your stomach feel right now?	4.0 ± 1.7	3.8 ± 2.6	3.8 ± 2.9	3.3 ± 3.1
How sick to your stomach do you feel right now?	1.6 ± 1.9	2.4 ± 2.6	1.7 ± 2.3	2.8 ± 3.3

## Data Availability

Data are contained and available within this manuscript.
